# Supplementary material for: Predicting visual function by interpreting a neuronal wiring diagram
Source: Nature. 2024 Oct 2;634(8032):113–23. doi: 10.1038/s41586-024-07953-5 (PMC11446822; doi:10.1038/s41586-024-07953-5)
Supplement: Supplementary file 6 — CRF and ERF predictions for individual TmY4 and TmY9 cells. Analogous to Supplementary Data 3, but for TmY target types. Shown are the top four monosynaptic pathways, the strongest pathway passing through each of the top ten intermediary types (ranking from Extended Data Fig. 7), and the trisynaptic pathway Tm1–TmY–Dm3–TmY (see the section entitled Prediction of spatial normalization). [file 41586_2024_7953_MOESM6_ESM.zip › DataS4/TmY9qΓèÑ/720575940622670836.pdf]

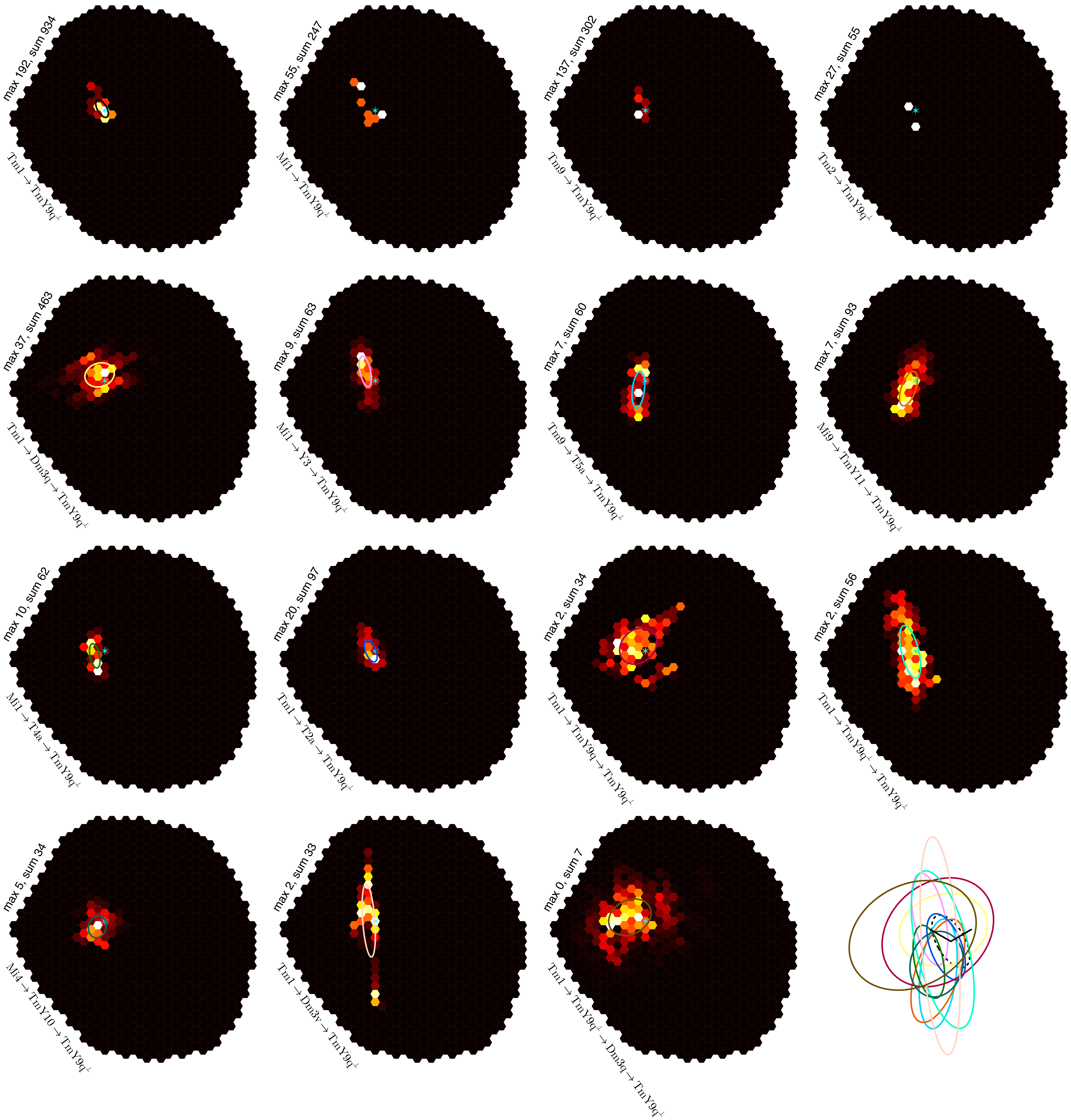
max 192, sum 934
Tm1 → TmY9q⊥
max 55, sum 247
Mi1 → TmY9q⊥
max 137, sum 302
Tm9 → TmY9q⊥
max 27, sum 55
Tm2 → TmY9q⊥
max 37, sum 463
Tm1 → Dm3q → TmY9q⊥
max 9, sum 63
Mi1 → Y3 → TmY9q⊥
max 7, sum 60
Tm9 → T5a → TmY9q⊥
max 7, sum 93
Mi9 → TmY11 → TmY9q⊥
max 10, sum 62
Mi1 → T4a → TmY9q⊥
max 20, sum 97
Tm1 → T2a → TmY9q⊥
max 2, sum 34
Tm1 → TmY9q → TmY9q⊥
max 2, sum 56
Tm1 → TmY9q⊥ → TmY9q⊥
max 5, sum 34
Mi4 → TmY10 → TmY9q⊥
max 2, sum 33
Tm1 → Dm3v → TmY9q⊥
max 0, sum 7
Tm1 → TmY9q⊥ → Dm3q → TmY9q⊥
